# Modelling seasonal household variation in harvested rainwater availability: a case study in Siaya County, Kenya

**DOI:** 10.1038/s41545-023-00247-9

**Published:** 2023-04-13

**Authors:** Weiyu Yu, Peggy Wanza, Emmah Kwoba, Thumbi Mwangi, Joseph Okotto-Okotto, Diogo Trajano Gomes da Silva, Jim A. Wright

**Affiliations:** 1 School of Ecological Technology and Engineering, Shanghai Institute of Technology, Fengxian campus, Shanghai, 201418 China; 2 School of Geography and Environmental Science, University of Southampton, Building 44, Highfield campus, Southampton, SO17 1BJ UK; 3 Centre for Global Health Research, Kenya Medical Research Institute, P.O. BOX 1578-1400, Kisian campus, Kisumu-Busia Highway, Kisumu, Kenya; 4 Paul G Allen School for Global Animal Health, Washington State University, Pullman, WA 99164-7090 USA; 5 Victoria Institute for Research on Environment and Development (VIRED) International, P.O. BOX 6423-40103, off Nairobi Road, Rabuor, Kenya; 6 Environmental and Public Health Research and Enterprise Group, School of Applied Sciences, University of Brighton, Cockcroft Building, Lewes Road, Brighton, BN2 4GJ UK

**Keywords:** Geography, Developing world, Water resources

## Abstract

Rainwater harvesting reliability, the proportion of days annually when rainwater demand is fully met, is challenging to estimate from cross-sectional household surveys that underpin international monitoring. This study investigated the use of a modelling approach that integrates household surveys with gridded precipitation data to evaluate rainwater harvesting reliability, using two local-scale household surveys in rural Siaya County, Kenya as an illustrative case study. We interviewed 234 households, administering a standard questionnaire that also identified the source of household stored drinking water. Logistic mixed effects models estimated stored rainwater availability from household and climatological variables, with random effects accounting for unobserved heterogeneity. Household rainwater availability was significantly associated with seasonality, storage capacity, and access to alternative improved water sources. Most households (95.1%) that consumed rainwater faced insufficient supply of rainwater available for potable needs throughout the year, with intermittencies during the short rains for most households with alternative improved sources. Although not significant, stored rainwater lasts longer for households whose only improved water source was rainwater (301.8 ± 40.2 days) compared to those having multiple improved sources (144.4 ± 63.7 days). Such modelling analysis could enable rainwater harvesting reliability estimation, and thereby national/international monitoring and targeted follow-up fieldwork to support rainwater harvesting.

## Introduction

Target 6.1 of the Sustainable Development Goals (SDGs) seeks to ‘achieve universal and equitable access to safe and affordable drinking water for all’^1^. Within this target, ‘access’ implies ‘sufficient water to meet domestic needs is reliably available close to home’^2^. The corresponding indicator proposed by the Joint Monitoring Programme for Water Supply, Sanitation and Hygiene (JMP) of the World Health Organization (WHO) and the United Nations Children’s Fund (UNICEF) builds on a previously established measure for monitoring the Millennium Development Goals (MDGs), namely, use of an improved drinking water source. The new indicator includes availability (‘available when needed’) as one of several criteria identifying ‘safely managed drinking water’, alongside accessibility (‘located on premises’) and quality (‘compliant with faecal and priority chemical standards’)^2^. Availability of drinking water was not only incorporated into the indicator for SDG monitoring as a normative human rights criterion^3^, it also significantly affects public health and wellbeing^4^ and supports economic, social and cultural development in many countries, including Kenya^5^.

Rainwater harvesting (RWH) is where rainfall runoff is collected on premises from surfaces, typically a roof catchment, and stored in a container or reservoir for drinking or other purposes. Being natural and decentralised, RWH offers good perceived quality, low environmental impact, health benefits, energy saving and convenience at household level^6,7^, and thus may improve rural drinking water availability in rural areas of developing countries where groundwater and surface water resources are limited or contaminated^8–10^. The WHO/UNICEF JMP has classified RWH as an ‘improved water source’ given its potential to deliver safe water^11^. Following the growing awareness of water conservation and sustainable developments alongside progressive stress on water resources, the dependence on RWH as a drinking water source is increasing globally^12^. However, given the seasonal and often unpredictable variations in precipitation, most household RWH systems are unable to provide a continuous supply of sufficient quantity through dry periods. For example, a previous study^5^ in eastern Kenya found that harvestable rainwater during the wet seasons could last for ~82 days, insufficient to cover dry season household water demand. Therefore, RWH often serves as an alternative or supplementary drinking water source^13^.

For RWH systems, availability is typically expressed as reliability, defined as the proportion of days per year that the system meets user needs. As a significant factor inhibiting rainwater use, the reliability of RWH systems can be calculated via a water balance modelling approach, in which a volume of collectable water is first calculated from the roof catchment area, daily rainfall, and a runoff coefficient^14^. When combined with storage tank capacity and household daily water demand estimates, collectable water volume can be used to estimate the proportion of days when demand is met and so measure reliability. Alongside assessment of household willingness to pay for upgraded RWH infrastructure, such calculations are often used to optimise storage capacity and roof catchment areas, so as to increase the reliability^14^.

**Fig. 1:**
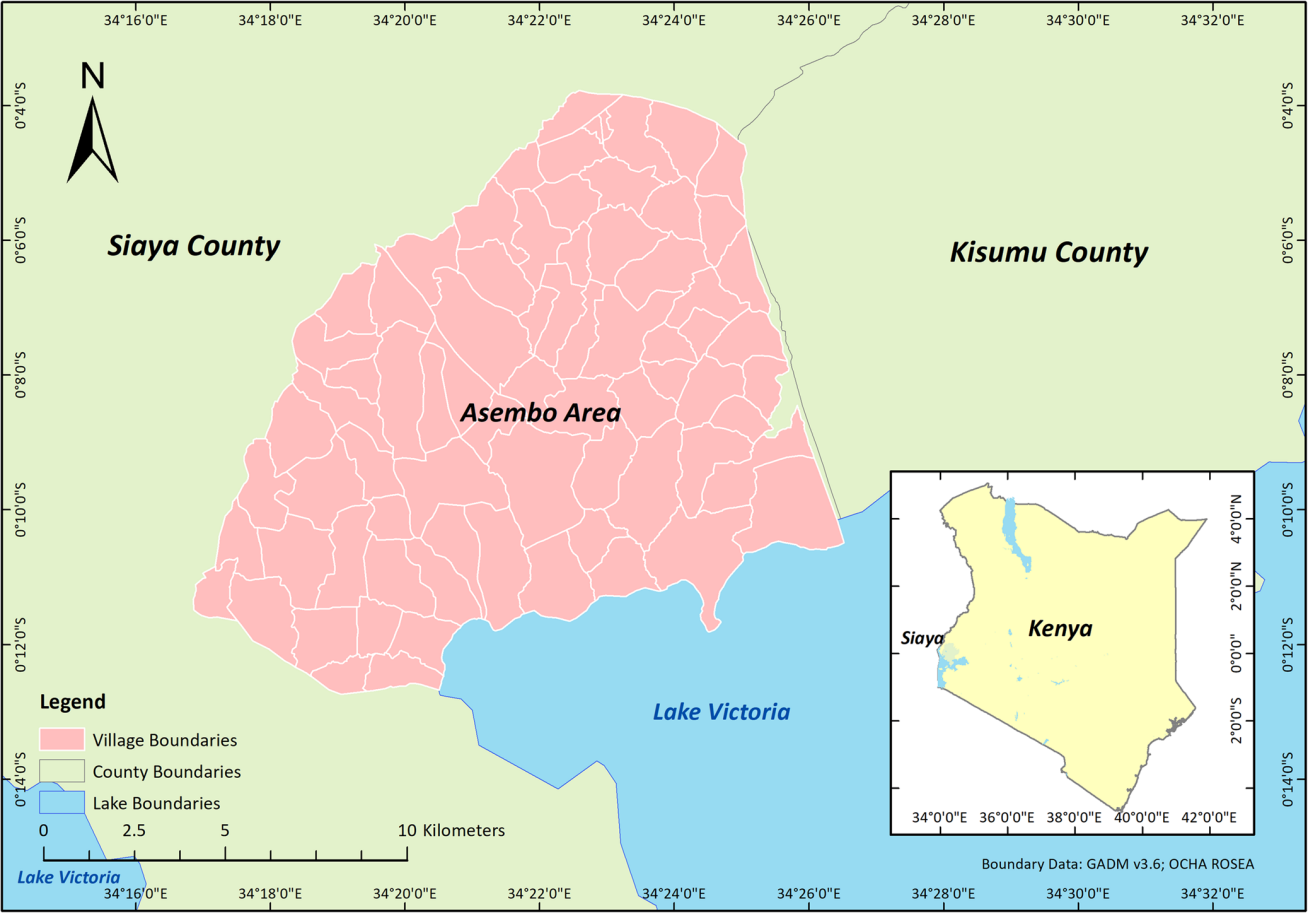
Map of the Asembo study area in Siaya County, western Kenya. The inset map shows the location of Siaya County (in green) in Kenya.

Understanding RWH reliability at the national level supports monitoring of SDG target 6.1 and spatial targeting of data collection to assess potential RWH system upgrades. However, limited survey implementation resources often restrict the content of multi-purpose, nationally representative household surveys frequently used for monitoring of SDG target 6.1 resources^15^. Since surveys such as the Demographic and Health Surveys (DHS) and Multiple Indicator Cluster Surveys (MICS) are multi-purpose and used to monitor multiple SDGs, their thematic content in any one sector such as Water, Sanitation and Hygiene (WASH) is restricted. The JMP have developed a core and expanded set of household survey questions for WASH^16^, but questions specific to RWH systems, such as storage tank capacity, roof catchment material and roof catchment area measurements, are thus necessarily not included. Our main objective in this study is therefore to explore whether the addition of two new expanded household survey questions, identifying the source and availability of a household’s stored drinking water on the day of the interview, would enable estimation of RWH reliability through a modelling framework. To evaluate this, we draw on household survey data from two local-scale projects in Asembo area, Siaya County, western Kenya (Fig. 1), namely the OneHealthWater (OHW) project and the Population-Based Animal Syndromic Surveillance (PBASS) project, which included these questions alongside other questions commonly found in national household surveys. We integrate these survey data with gridded precipitation data that would be scalable to national level. As a secondary objective, we also assess potential differences in rainwater availability between households with and without access to alternative improved water sources.

## Results

### Household characteristics

**Fig. 2:**
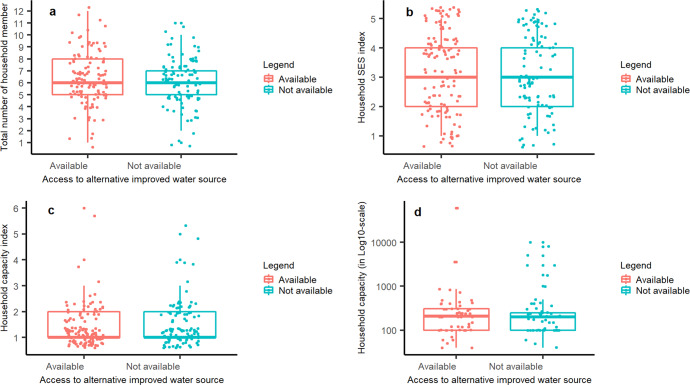
Boxplots showing household characteristics by access to alternative improved water sources. Separate graphs are presented for different household characteristics: **a** total number of household members. **b** household socio-economic status characterised by an SES index. **c** household capacity index. **d** household rainwater storage capacity in Log10-scale. The bottom and top of the box are the 25th and 75th percentiles respectively, whilst the thick line that divides the box represents the median value; the superimposed coloured dots show individual households.

A total of 234 households were interviewed, of which four households that did not participate in the second round of the OHW survey (where household water storage reservoir used exclusively for storing rainwater was identified), five households that did not report rainwater as a drinking water source, and one household that did not report any water storage were then excluded. The final sample for model fitting (hereinafter referred to as the ‘training data’) therefore consists of 448 observations from 224 households, whilst the sample used for model performance evaluation (hereinafter referred to as the ‘test data’) consists of 100 observations from 100 households. The majority (66.07%) of the study households interviewed rely on small vessels with storage capacities of less than 150 litres. Detailed summaries of household characteristics potentially affecting household stored rainwater availability at the time of the interview are shown in Table 1. There were no significant differences in household characteristics between households with and without alternative improved water sources (see Fig. 2).

**Table 1:** Characteristics of study households (*n* = 224).

Household characteristics	Data summary
Household total capacity index	
1 Very small (<150 litres)	148 (66.07)
2 Small (150–500 litres)	64 (28.57)
3 Moderate (500–1800 litres)	5 (2.23)
4 Large (1800–5000 litres)	4 (1.79)
5 Very large (5000–10,000 litres)	2 (0.89)
6 Extremely large (>10,000 litres)	1 (0.45)
Household size (number of persons)	6.28 (± 2.06)
Household SES quintile	
1st quintile – poorest	29 (12.95)
2nd quintile	56 (25.00)
3rd quintile	36 (16.07)
4th quintile	54 (24.11)
5th quintile – least poor	49 (21.88)
Alternative improved water source	
Available	123 (54.91)
Not available	101 (45.09)

All continuous data are reported as mean (± standard deviation); whilst all ordinal data are reported as total number (%) by each class.

### Local climatological seasonality

**Fig. 3:**
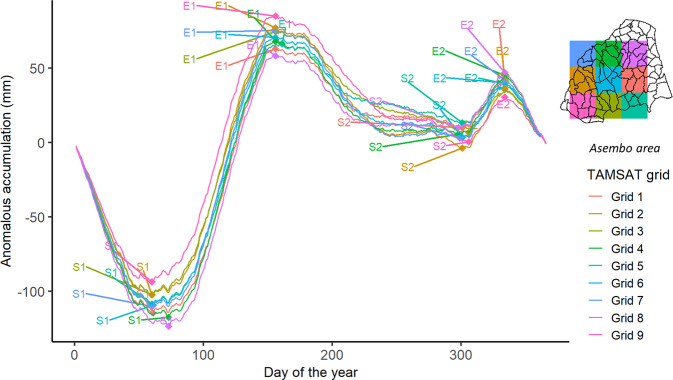
Climatological anomalous rainfall accumulation curves for nine 4 km × 4 km TAMSAT grids covering the study area. ‘S1’ and ‘E1’ respectively represent the start and end of the long rainy season for each TAMSAT grid, whilst S2 and E2 are the start and end of the short rainy season respectively for each grid. The long dry periods are between ‘E1’ and ‘S2’; whilst the short dry periods are between ‘E2’ and ‘S1’.

Figure 3 shows the climatological anomalous rainfall accumulation curve produced for nine 4 km × 4 km TAMSAT grid cells covering the Asembo study area, Siaya County, Kenya. The rainfall patterns are bimodal for all nine grid cells, but with local variations in climatological seasonality apparent. For example, for Grid 9, the long rainy season begins around late February or early March (day 60) and ends around early June (day 156), whilst the short rainy season runs from early to late November (day 306–day 334). In contrast, for Grid 8, the long rainy season begins around mid-March (day 73) and ends around early June (day 156), whilst the short rainy season runs from late October (day 298) to late November (day 334). After the long climatological rainy season, households living within Grid 9 will likely experience a much longer period (approx. two weeks) of climatological dry season than those living within Grid 8 before reaching the short climatological rainy season. Additionally, the climatological short rainy season of Grid 9 is also slightly shorter (8 days) than that of Grid 8.

### Model results

**Fig. 4:**
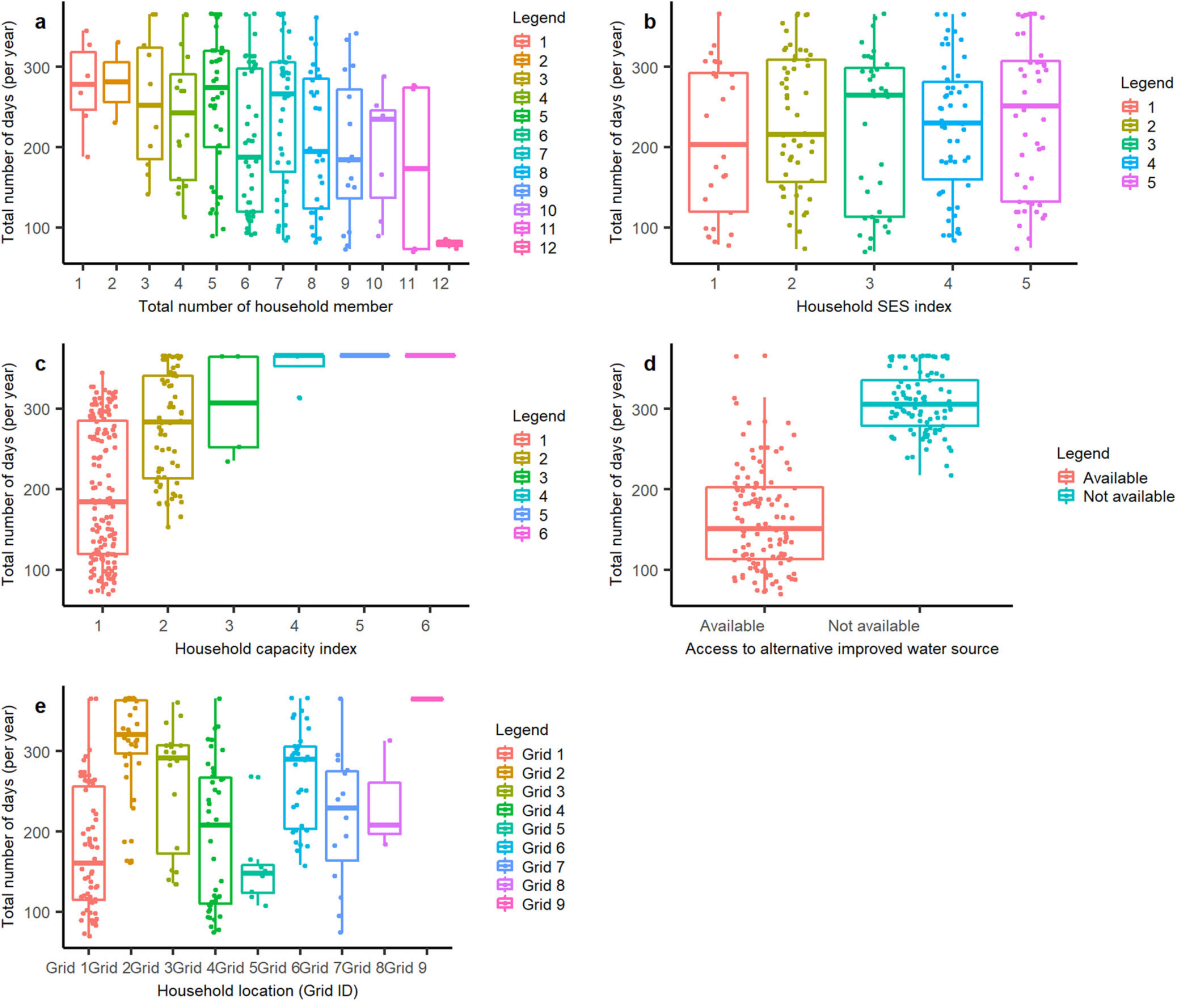
Boxplots showing total number of days annually that study households have stored rainwater available, broken down by household characteristics. Separate graphs are presented for different household characteristics: **a** total number of household members. **b** household socio-economic status characterised by an SES index. **c** household capacity index. **d** household access to an alternative improved water source. **e** household location defined by TAMSAT grid ID. The bottom and top of the box are the 25th and 75th percentiles respectively, whilst the thick line that divides the box represents the median value; the superimposed coloured dots show individual households.

**Fig. 5:**
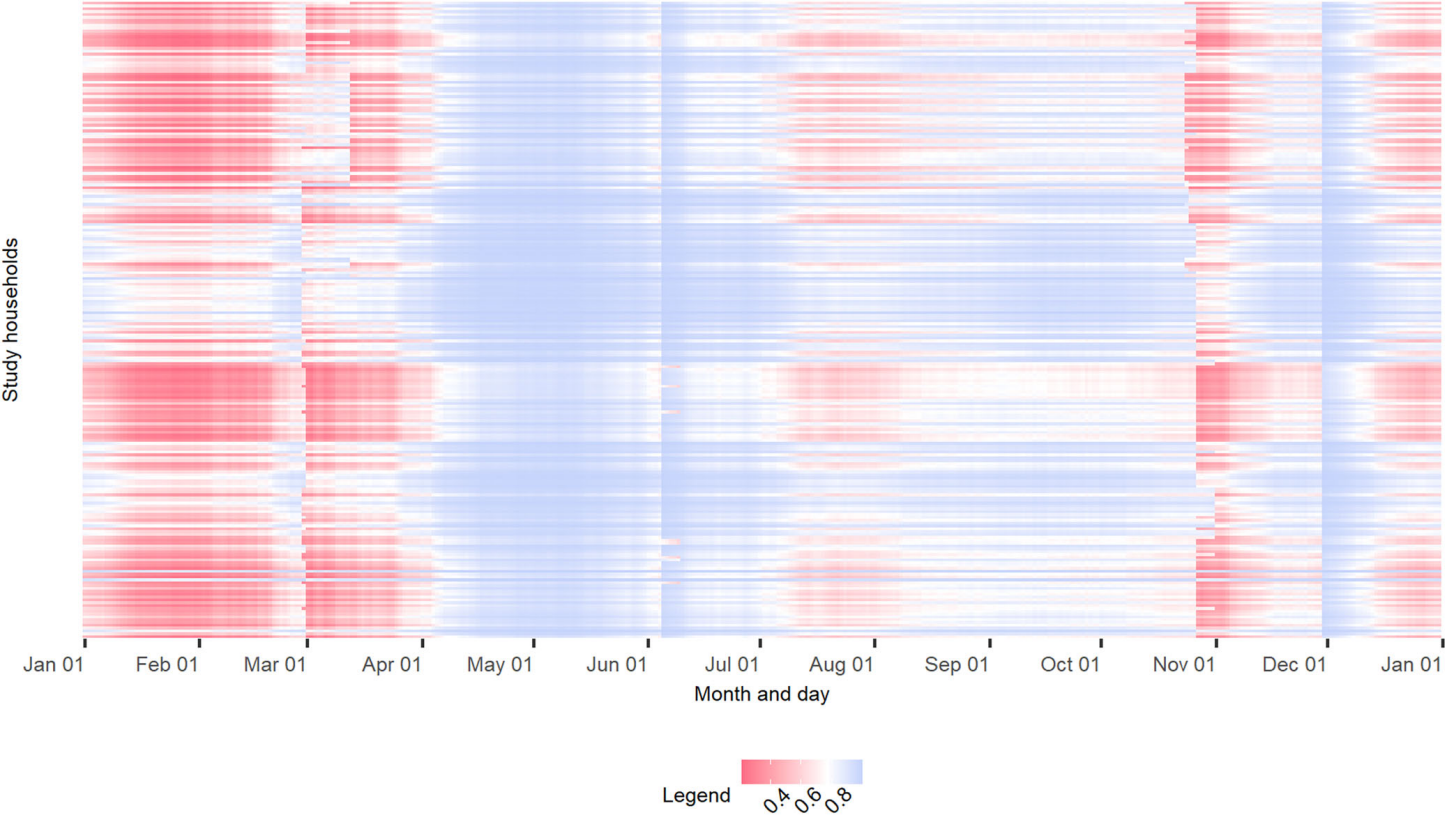
Heat map of predicted availability of rainwater stored at home. Each column represents a day (of a year), and each row represents one of the 224 study households. Predicted probability of having stored rainwater was scaled from red (NOT AVAILABLE: smaller than the optimal cutoff value of 0.767 (in white), selected by maximising the sum of sensitivity and specificity) to blue (AVAILABLE: greater than 0.767) colours.

**Fig. 6:**
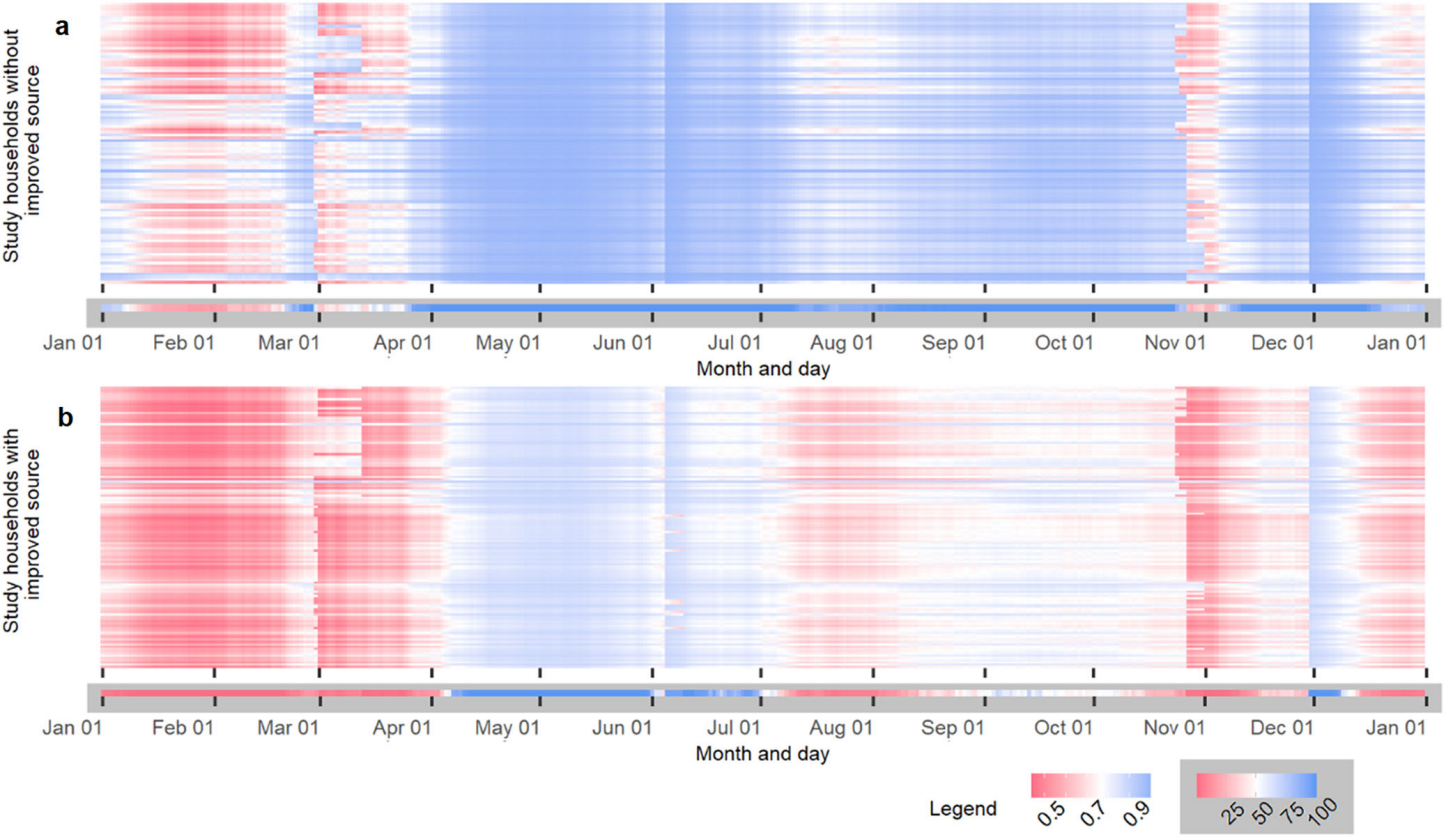
Heat map of predicted availability of rainwater stored at home, broken down by household access to alternative improved water sources. In the upper heat maps, each column represents a day (of a year), and each row represents a study household. Predicted probability was scaled from red (NOT AVAILABLE: smaller than the optimal cutoff value of 0.767 (in white), selected by maximising the sum of sensitivity and specificity) to blue (AVAILABLE: greater than 0.767). The lower inset bars (with a grey background) show the percentage of all households without (**a**; *n* = 101) and with (**b**; *n* = 123) alternative improved water sources that had harvested rainwater available at home on a given day of a year, scaled from red (less than the 50 percent threshold as shown in white) to blue (greater than 50 percent).

In this study, rainfall totals for 14 days preceding the survey gave the most parsimonious model fit (AIC = 359.3) among the 30 tested preceding periods. The mixed model displayed good performance (test AUC = 0.858), which suggests that it explained the majority of variance in the data. As summarised in Table 2, time-varying variables depicting precipitation and seasonality and time-invariant variables depicting household rainwater storage capacity and access to alternative improved water sources were significantly associated with availability of stored rainwater. Figure 4 illustrates predicted rainwater reliability (total number of days annually per household with stored rainwater available), broken down by four household characteristics employed in our model. The vast majority (213 out of 224; 95.1%) of the study households do not have rainwater available at home throughout a year. Figure 5 shows the predicted distribution of household rainwater availability, which highlights the periods within the year when households run out of rainwater at home. Among households without alternative improved drinking water sources, predicted rainwater availability varies considerably by household context (301.8 ± 40.2 days), with the best-served households having rainwater available throughout the whole year, whilst the worst have rainwater available for approximately 203 days per year. For households with access to alternative improved water sources, in general, relatively shorter periods of rainwater availability can be observed in comparison with the households without alternative improved sources (144.4 ± 63.7 days; see Fig. 6). Among the 123 households with alternative improved water sources, 88 (71.5%) are predicted to run out of stored rainwater for more than half a year. The lowest predicted number of days annually that a household with alternative improved sources has stored rainwater available is 64 days. In general, our model predicts periodic seasonal shortages of household rainwater availability (Figs. 5, 6), broadly in line with climatological seasonality, with most households using RWH as their main source having stored rainwater available during both the long and short rainy seasons (Fig. 6a). In contrast, for many households with alternative improved drinking water sources, intermittent rainwater availability can be observed in the short rainy season (Fig. 6b). Detailed distribution maps of predicted number of households (in total, and broken down by access to alternative improved water sources) with 95% prediction intervals are shown in Supplementary Information 1.

**Table 2:** Results of the logistic mixed effects model of household rainwater availability using rainfall totals for 9 days preceding the survey.

Covariate	Estimate	95% confidence interval	*P* value
Cumulative rainfall for 14 days prior to the survey date	0.064	(0.045, 0.083)	<0.001***
Days since the last wet season	−0.004	(−0.021, 0.013)	0.637
Dry season	1.942	(0.692, 3.193)	0.002**
Total number of household members	−0.059	(−0.205, 0.088)	0.431
Household SES index	0.032	(−0.186, 0.250)	0.774
Household total capacity index	0.477	(0.037, 0.918)	0.034*
Access to alternative improved water source(s)	−1.003	(−1.650, −0.356)	0.002**

*p < 0.05; **p < 0.01; ***p < 0.001.

## Discussion

By integrating household survey and gridded precipitation data, this study demonstrated how two extended survey questions can be used to model RWH reliability with good predictive performance. These two questions could potentially be added to a national household survey such as a DHS in a country where RWH is practiced widely, to enable RWH reliability modelling at the national level. The analysis provides evidence-based insights into RWH reliability not only for monitoring of progress towards SDG target 6.1, but also for supporting RWH system upgrades. Our local-scale modelling analysis suggests that most households in Siaya County, western Kenya that consume rainwater face an insufficient supply of rainwater available for potable use throughout the year. Our finding is broadly in line with a previous study in Embu County, eastern Kenya^5^ where harvestable rainwater showed a bimodal pattern, sufficient only for ~82 days in total during the long (from March to May) and short (from October to December) rains. Whilst RWH is classified as an improved water source by WHO and UNICEF, our modelling indicates that the proportion of population using ‘safely managed’ drinking water sources could often be substantially overestimated for this group, given that RWH remains the only improved drinking water source for many of these households.

To incorporate availability (‘available when needed’) into SDG monitoring of drinking water, the JMP have developed a core and expanded set of questions for household surveys^16^. The core question W5 (i.e. ‘In the last month, has there been any time when your household did not have sufficient quantities of drinking water when needed?’) has been included in the recent DHS and MICS household surveys specifically for measuring drinking water availability^17,18^. Whilst this question is only asked of households using piped water, borehole, or public tap/standpipe as their main water source in the DHS^17^, it is asked of households using all drinking water source types in MICS household surveys, so as to calculate the MICS indicator WS.3 (i.e. percentage of household members with drinking water available when needed)^19^. It uses the occurrence of water insufficiency ‘in the last month’ preceding the interview as an indicator of availability of sufficient drinking water when needed. However, since our study found a bimodal pattern in distribution of household rainwater availability (Fig. 6a), the occurrence of water insufficiency ‘in the last month’ may not reflect annual reliability of RWH. For example, households relying on RWH may likely run out of rainwater only during dry seasons, but interviews conducted in the late rainy seasons would not capture this shortage, thus potentially over-estimating RWH coverage, and in turn, over-estimating ‘safely managed’ drinking water coverage in settings where harvested rainwater is free from faecal and priority chemical contaminants. When large-scale household surveys often compress fieldwork into a relatively short period of time due to reasons such as resource availability or project schedule, attention must be made in survey planning and implementation to ensure any seasonality effects are evaluated^20^. Given incorporation of the two additional questions in a household survey, the modelling analysis as demonstrated in this study could provide an alternative means of estimating RWH reliability, thereby avoiding these pitfalls in estimating RWH availability. Such an analysis could also help in planning the seasonal timing of household survey implementation and interpreting past household survey findings in light of likely seasonal RWH household behaviours.

The outputs of this study reveal apparent differences in rainwater availability between households with and without alternative improved water sources. In general, stored rainwater tends to last longer for households who use RWH as their only improved water source (as shown in Fig. 4d), despite a lack of significant differences in other characteristics between these two groups (Fig. 2). Many such households would need to revert to using surface waters such as Lake Victoria, streams, and ponds, a dilemma faced by many other users of intermittent improved sources globally according to systematic review evidence^21^. In other countries such as the Solomon Islands^22^, rationing behaviours have been reported among RWH users when faced with scarcity. It, therefore, seems plausible that this finding reflects rationing of harvested rainwater by households lacking access to an alternative improved source. In contrast, households with multiple improved source options may temporarily substitute rainwater for drinking water from other improved sources when available, since rainwater is free-of-charge and often considered safe and palatable by Siaya residents^23^. Such potential behavioural and consumption patterns may explain the apparent intermittencies in RWH availability for households with alternative improved sources during the short rains in Fig. 6b. Households with multiple improved water sources may store rainwater from April to late June, and then practice source-switching frequently during the latter short rains from July to October (Fig. 6b). Such source-switching behaviour allows these households to avoid overreliance and depletion of a single valuable source^24^. In related fieldwork in Siaya County^25^, we found some households had designed storage tanks with both piped and rainwater intakes, thereby adapting to water scarcity but potentially increasing exposure to water-borne pathogens^26^. In contrast, households without alternative improved water sources rely mostly on stored rainwater during much of the short dry season and short rains from April to October. Outside of these periods, such households may be forced to use unimproved sources and thus be exposed to faecally contaminated drinking water. This suggests that having data concerning secondary or seasonal water sources would be important, were our approach to rainwater harvesting reliability estimation to be scaled up nationally or internationally.

RWH is a means of improving drinking water availability in a wide range of climatic and socio-economic environments, particularly in rural areas of developing countries where groundwater and surface water sources are often contaminated, limited or otherwise have low potential^8–10^. Despite RWH often being considered a supplementary or secondary source, it sometimes can also be reported as a primary drinking water source^21^. In this case study, our findings reflect that most Siaya residents without access to alternative improved sources may use RWH as their primary drinking water source, given that RWH could potentially meet their needs for approximately 84.2% of days of a year in average. In contrast, RWH is likely used as a supplementary or secondary source to bolster resilience in households with multiple options of improved water sources, since RWH could only cover their needs for 44.0% of days of a year in average. Previous studies revealed high demand and preference for rainwater not only in Kenya^27^, but also in other parts of the world^28–30^. Particularly where GPS coordinates are collected as part of a household survey, there is potential to spatially target RWH resources. Examples of such support could include local willingness-to-pay assessments for RWH improvements, localised RWH water quality testing, technical support for RWH upgrades, and even targeted marketing of newer innovations, such as a cell phone app recently developed to assess the appropriateness of RWH configuration^31^.

Our modelling analysis also highlights the value of collecting GPS coordinates in household surveys. Previous studies have illustrated the utility of georeferenced household survey data in generating spatially explicit estimates of WASH indicators^32,33^. This study, on the other hand, shows the importance of including GPS coordinates, so as to enable spatial integration of household survey with other data products for further analysis. However, for large-scale nationally representative household surveys, GPS coordinates are often released with random displacement to protect respondent confidentiality. For example, DHS cluster coordinates are randomly displaced by up to 2 km for urban locations and up to 5~10 km for rural areas^34^. This may undermine their utility in local-scale analysis and may introduce uncertainty when integrating with high-resolution gridded data products such as TAMSAT.

This study is subject to several limitations as follows: firstly, whilst nationally representative household surveys such as DHS and MICS are often conducted in a wider variety of contexts, our study was longitudinal, not cross-sectional, and thus was merely representative of the study villages in Asembo area, Siaya County, western Kenya, potentially limiting its generalisability. Secondly, this study drew upon two local-scale multi-purpose household surveys and incorporated a limited number of questions related to household rainwater harvesting or water demand into a simplified RWH model. However, a typical RWH reliability assessment includes measurement of roof catchment area and RWH storage capacity, alongside household water demand estimation^35^. Since roof catchment area would be difficult and expensive to measure through a national household survey campaign, we did not include it in our study, so this RWH parameter is omitted from our model. Thirdly, rather than having specific RWH systems, many households in Siaya County harvested small quantities of rainwater with vessels of any kind available (e.g., 20-litre jerry-cans, pails, pots, basins, etc.), which presented difficulties when estimating rainwater storage capacity. Our household survey therefore may underestimate actual household rainwater storage capacity, which in turn affects our analysis and outputs. Fourthly, assessing household water demand is notoriously difficult when planning RWH in rural areas of developing countries, given multiple source use and the lack of reliable, affordable metering systems^22^. Here, we used household size and socio-economic status as proxy indicators of household water demand, but depending on national context and household survey content, other variables such as household livelihoods or adult member educational status could be explored instead^36^. Additionally, due to lack of published evidence for model parameterisation, we used AIC statistics for successive logistic regression models fitted using total rainfall of periods between one and 30 days preceding the survey to identify the optimal period duration for modelling. The optimal period for TAMSAT, pooling all households in this case study, was 14 days, but this varied depending on precipitation data used and for sub-groups of households. This estimated period may reflect storage times for harvesting rainwater and household storage capacity, but should be interpreted with caution given the estimated period’s sensitivity to input data and model selection metrics. Finally, instead of using station data, this case study adopted a gridded precipitation data product to illustrate a form of climate data that would be scalable to national level. However, such gridded data often has a coarse spatial resolution for a small-scale study area and may not reflect the actual local variation in rainfall.

The growing availability of daily gridded climate data plus expanded WASH content in georeferenced household surveys could provide an opportunity for quantification of RWH reliability through a simplified national-scale model. However, this would require the addition of a new pair of survey questions concerning the availability and source of water stored in the home at the time of the interview. We, therefore, recommend that household surveys in countries where RWH is widespread and where household preference for RWH technology is high could incorporate such a question pair. This would enable estimation of RWH reliability and thereby provide evidence for spatially targeted RWH support and richer monitoring insights. In scaling up this approach, we also recommend avoiding study countries in regions that have sparse or unreliable rainfall gauges and/or where intra-seasonal variability is poorly captured by high-resolution gridded rainfall data products. An alternative future study design could collect more detailed household survey data on roof catchment area, household water demand, and rainwater storage capacity, alongside recording the availability of stored rainwater in the home. Enumerator time spent collecting these data could also be recorded. This would enable the systematic assessment of the trade-off between survey implementation costs and successive improvements in the predictive performance of RWH reliability estimates through greater model complexity and the inclusion of more household variables.

## Methods

### Study site

The study area of Asembo, Siaya County is in rural western Kenya on the shores of Lake Victoria (Fig. 1). Rainfall in Siaya County is bimodal with a long rainy season between March and June, and a short rainy season between September and December. Influenced by altitude, annual rainfall is ~800–2000 mm^37^. Resident communities suffer high poverty levels^38^ and a high burden of infectious diseases^39^ concurrently. In Siaya County, rainwater is widely considered the safest and most preferred drinking water source by the local communities according to their religious perceptions^23^. Approximately 32% of Asembo households consumed rainwater^40^.

### Study design and data acquisition

This study collected data primarily from a household survey module in the OneHealthWater (OHW) project, designed to examine livestock-related microbial contamination of household stored water^41^. The OHW household survey captured several household characteristics that may affect rainwater harvesting and storage. In total, 234 households were selected at random from 1500 households participating in an ongoing Population-Based Animal Syndromic Surveillance (PBASS)^40^ after seeking their informed consent. Participants were interviewed twice during 2018–2020 in both wet and dry seasons to collect information on domestic water and household conditions and behaviours presenting contamination hazards through close-ended questions. 234 households were interviewed from 12th March to 24th May 2018, and 230 of these households were interviewed from 20th November 2018 to 18th february 2019, excluding four households who were unavailable. Data collection was performed using a smartphone-based app, CommCare^®^ (https://www.dimagi.com/commcare/). To measure the availability and source of a household’s stored drinking water at the time of interview, we asked households “do you have any stored water now?” We then asked those households answering yes “Where did the water stored in this container come from?” (see Supplementary Information 2).

The OHW household survey data was then integrated with the PBASS data stream, which also contains household-level data on socio-economic status, human health and animal health^40,42^. We restricted household characteristics included in modelling to those plausibly related to water demand or rainwater storage practices and commonly found in national household surveys such as the DHS. We included household size alongside wealth quintiles derived from a socio-economic status (SES) index^43^ for PBASS households, since both may affect household water demand^36^ and since wealth quintiles are routinely calculated for national household surveys such as the DHS^44^. In addition, wealth may also potentially affect a household’s capacity for storing water and ability to construct and maintain an appropriate harvesting system^28^.

To illustrate a means of integrating household survey and climate data that would be scalable to national level, we derived daily rainfall data from the Tropical Applications of Meteorology using SATellite data and ground-based observations (TAMSAT) version 3.1 dataset^45^, which provides a high-resolution (0.0375 degree, ~4 km) satellite-based precipitation estimates from 1983 to the delayed present for Africa at daily to seasonal time intervals. We chose this precipitation dataset for its gridded spatial representation that would be scalable to national-level household survey analyses, for its higher spatial resolution, and comparatively higher accuracy in depicting sub-seasonal rainfall in eastern Africa^46^. Firstly, we extracted TAMSAT daily rainfall from 1st January 1983 to 31st December 2020 (i.e., the last year of our fieldwork) at each household location in order to explore the long-term locational variation in climatological seasonality. In preference to other methods, such as those that define the dry season as the period when potential evapotranspiration exceeds precipitation^47^, the climatological anomalous rainfall accumulation method developed by Liebmann et al.^48,49^ was employed to identify the onset and cessation of rainy season, given its suitability for regions experiencing long and short rains^50^. For each household location, survey dates were then classified as wet (rainy) or dry (low rainfall) season accordingly. Anomalous rainfall accumulation is the sum of the daily precipitation minus the long-term annually-averaged daily precipitation. For each location, an anomalous rainfall accumulation curve can be created by plotting the anomalous accumulation against the day of a year, where an upward slope can be used to define the local climatological wet season, whilst a downward slope can be used to determine the local dry season. The total number of days since the end of the last rainy season was then calculated for each household survey visit, setting this to zero if the household was visited during a rainy season. In addition, for each household location, cumulative total rainfall was calculated for all periods between one and 30 days prior to the household survey date.

Many households used small containers to harvest rainwater, such as 20-litre jerry-cans, even though they lacked more sophisticated rainwater storage systems. Household capacity was therefore converted to an ordinal variable with six levels using k-means clustering (referred to as a household capacity index) in order to avoid numerical difficulties in statistical analysis.

For validation purposes, we further adopted the same two questions as a part of the subsequent 2019–2020 PBASS routine data collection for the same 234 households who participated in the OHW study. This data was then integrated with the OHW and the TAMSAT data to create the ‘test data’ for model performance evaluation. Due to the impact of the coronavirus 2019 (COVID-19) pandemic, the PBASS fieldwork was suspended part-way through the household survey campaign, meaning that only a subset of households was interviewed. In total, 104 households were successfully interviewed between 11 Nov 2019 and 19 March 2020 before the COVID-19 lockdown.

### Data analysis

The availability of rainwater stored at household level was modelled as a function of the identified environmental and socio-economic factors (see Table 3) as explanatory variables. We assumed that household storage of rainwater for drinking and domestic use follows a Bernoulli distribution, which takes a value of one when the household has stored rainwater at home for drinking on a specific day and a value of zero otherwise. Successive logistic regression models were fitted using total rainfall of periods between one and 30 days preceding the survey alongside other explanatory variables, with the best model chosen based on the Akaike Information Criterion (AIC)^51^. We utilised logistic mixed effects models with random effects selected based on principal component analysis (PCA) to account for unobserved heterogeneity among 4 km × 4 km TAMSAT grids and among households within TAMSAT grids. The pre-processed household survey data integrated with TAMSAT (i.e. the ‘training data’) was used to fit the models, whilst the ‘test data’ was used to evaluate the model performance. The Area Under the Receiver Operator Curve (AUC)^52^ calculated from the ‘test data’, with a range of 0.5 to 1, was used to evaluate model performance. The model is considered a perfect predictor for the outcome when the AUC value is 1.0, or has no predictive value when the AUC value is 0.5.

**Table 3:** Derived variables relevant to household use of potable rainwater harvesting.

Variable name	Type	Description	Data source
Rainwater stored	Time varying	The availability of rainwater stored for domestic use on the interview day (the outcome variable) reported by the participant who was asked “Do you have any stored water now?” and “Where did the water stored in this container come from?”	OHW; PBASS
Rainwater storage capacity	Time invariant	Total capacity (in litres) for household rainwater storage (exclusively), only captured in the second OHW survey visit.	OHW
Household size	Time invariant	The total number of household members.	PBASS
Household socio-economic status	Time invariant	A SES index showing wealth quintile rankings of households (from 1 = poorest to 5 = least poor).	PBASS
Alternative improved water source	Time invariant	Whether the household has any type of improved drinking water source other than rainwater.	OHW
Cumulative rainfall for 1–30 day(s) prior to the survey date	Time varying	Total rainfall (in mm) calculated successively from one day to 30 days prior to the household survey date.	TAMSAT
Seasonality	Time varying	The seasonality (i.e. either wet or dry season) of a household survey date	TAMSAT
Days since the last wet season	Time varying	The total number of days since the end of last wet season, calculated for each household and survey date.	TAMSAT

To better understand the pattern of seasonal changes in household rainwater source availability, for each participating household, we simulated daily availability of household stored rainwater based on long-term averages (1st January 1983–31st December 2020) of daily precipitation. The optimal cutoff value for predicted probability of having stored rainwater was selected by maximising the sum of sensitivity and specificity^53^. All data analyses were carried out in R 3.5.2^54^.

### Ethics and inclusion statement

P.W., T.M., J.O.O., and E.K. are all researchers based in Kenya, where fieldwork took place. As we make clear in our author contributions statement, this team of authors were integrally involved throughout the research process. J.O.O. works for VIRED International, a research and implementation NGO in Western Kenya and was an integral part of the research team. He led community engagement and feedback meetings following the project. Capacity-building plans for local researchers were discussed, with sharing of project-relevant expertise between the UK and Kenya. J.W. provided additional geospatial co-supervisory for TM’s PhD student M.N. working on a related topic. This research would not have been restricted or prohibited in Kenya. The research was approved by the Scientific and Ethical Review Committee of the Kenya Medical Research Institute. There are no specific animal welfare, environmental protection, or biorisk regulations that affect the conduct of this study and no significant risk to participants, as per the ethical review and approval process. We put in place health and safety measures for the whole research team, such as having health and safety reporting as a standing agenda item at project meetings. Citations of locally and regionally relevant research are included in our manuscript, e.g., ^37,38,40,42,43^.

### Supplementary information

Supplementary Information

## Data Availability

The data that support the findings of this study are available from the publicly accessible FigShare repository at: 10.6084/m9.figshare.21975695.

## Code availability

The analysis code for this study is available in FigShare and can be accessed via this link: 10.6084/m9.figshare.21975695.
