# Supplementary material for: Modelling seasonal household variation in harvested rainwater availability: a case study in Siaya County, Kenya
Source: NPJ Clean Water. 2023 Apr 13;6(1):32. doi: 10.1038/s41545-023-00247-9 (PMC10099009; doi:10.1038/s41545-023-00247-9)
Supplement: Supplementary file 1 — Supplementary Information [file 41545_2023_247_MOESM1_ESM.pdf]

Supplementary Information 1: Line Charts Showing the Distributions of Predicted Number of Households with 95% Prediction Intervals

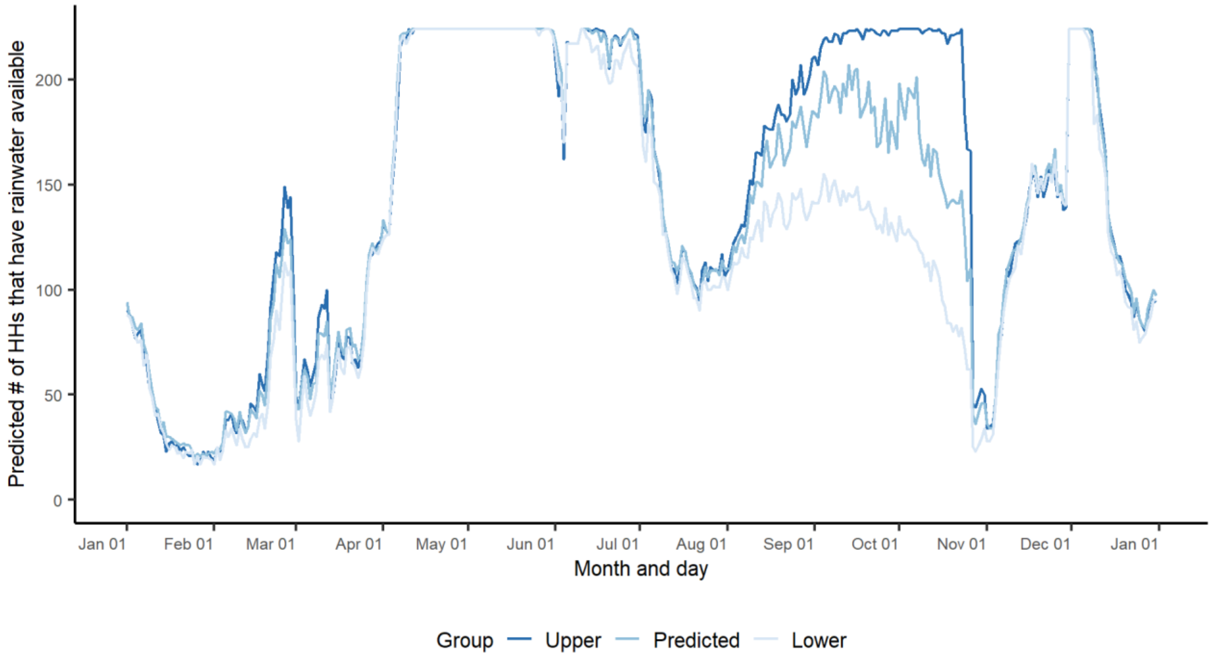

Supplementary Figure 1. Line chart showing how the predicted number of households that have harvested rainwater available ('Predicted') changes over time with 95% prediction interval ('Upper' and 'Lower').

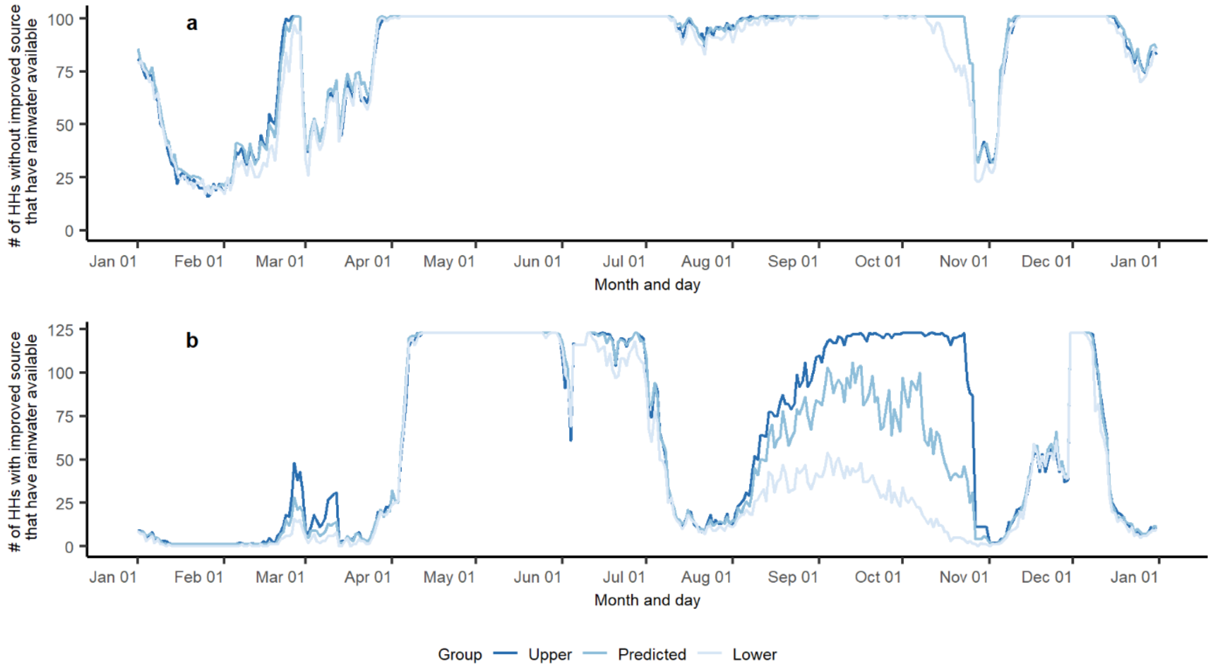

Supplementary Figure 2. Line charts showing how the predicted number of households without (a) and with (b) alternative improved water sources that have harvested rainwater available ('Predicted') changes over time with 95% prediction interval ('Upper' and 'Lower').

Supplementary Information 2: Expanded Questions Identifying the Source of a Household's Stored Drinking Water

Supplementary Question 1: (Q8) Do you have any water stored now?

Yes........................1

No.........................0

Supplementary Question 2: (Q14a) Where did the water stored in this container come from?

W1. Piped water into dwelling..1

W2. Piped to yard or plot...2

W3. Public tap or standpipe...3

W4. Tubewell or borehole...4

W5. Protected well...5

W6. Unprotected well..6

W7. Protected spring...7

W7A. Unprotected spring..8

W8. Rainwater..9

W9. Bottled water...10

W10. Cart with small tank...11

W11. Tanker truck..12

W12A. Free-flowing river or stream.....................................13

W12B. Stagnant water in dam, pond or lake......................14

W12C. Canal or irrigation channel...15

Other: Specify__________...20
